# Crystal structure and optical properties of fused-ring chalcone (*E*)-3-(anthracen-9-yl)-1-(4-nitro­phen­yl)prop-2-en-1-one

**DOI:** 10.1107/S2056989019005243

**Published:** 2019-04-25

**Authors:** Muhamad Fikri Zaini, Ibrahim Abdul Razak, Wan Mohd Khairul, Suhana Arshad

**Affiliations:** a X-ray Crystallography Unit, School of Physics, Universiti Sains Malaysia, 11800 USM, Penang, Malaysia; b School of Fundamental Science, Universiti Malaysia Terengganu, 21030, Kuala Terengganu, Terengganu, Malaysia

**Keywords:** chalcone, crystal structure, DFT, Hirshfeld surface, UV–Vis, HOMO–LUMO

## Abstract

The title compound, C_23_H_15_NO_3_, adopts an *s-cis* conformation with respect to the ethyl­ene C=C and carbonyl C=O double bonds in the enone unit. The mol­ecule is significantly twisted with a dihedral angle of 48.63 (14)° between the anthracene ring system and the benzene ring. In the crystal, mol­ecules are linked into inversion dimers with an *R*_2_^2^(10) graph-set motif *via* pairs of C—H⋯O hydrogen bonds. The inter­molecular inter­actions were analysed and qu­anti­fied by Hirshfeld surface analysis. The mol­ecular structure was optimized and a small HOMO–LUMO energy gap of 2.55 eV was obtained using the DFT method at the B3LYP/6–311 G++(d,p) level of theory. This value is in close agreement with the experimental value of 2.52 eV obtained from the UV–vis analysis. The crystal used was a two-component merohedral twin with a refined ratio of 0.1996 (16):0.8004 (16).

## Abstract 2

The title chalcone derivative adopts an *s-cis* conformation with respect to the enone fragment and is non-planar with a dihedral angle of 48.63 (14)° between the anthracene ring system and the nitro­benzene ring. In the crystal, mol­ecules are linked into inversion dimers with an 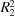
(10) graph-set motif *via* pairs of inter­molecular C—H⋯O hydrogen bonds.

## Chemical context   

Conjugated organic mol­ecules with multiple fused aromatic rings have attracted a great deal of inter­est from researchers because of their excellent performance in organic semiconductor devices (Gu *et al.*, 2015 ▸). These organic mol­ecules with a delocalized π-system represent attractive targets for applications in light-emitting diodes. In addition, the selection of the organic π-system with an electron donor (*D*) and an electron acceptor (*A*) is important because it exhibits an essential role in charge transfer in the mol­ecule, where the aromatic groups may lead to delocalization of electronic charge distribution, imparting higher polarization of the push–pull configuration and generation of a mol­ecular dipole (Bureš, 2014 ▸). An organic chalcone derivative with a π-conjugated system provides a large transfer axis with appropriate substituent groups on both terminal aromatic rings. The chalcone π-bridge consists of a α,β-unsaturated carbonyl unit which is responsible for intra­molecular charge transfer. From the previous studies by Xu *et al.* (2015 ▸), the introduction of fused aromatic rings into the push–pull system could lead to enhanced carrier mobility and a lower band gap. In a continuation of our previous work on the effect of a fused-ring substituent, *i.e.* naphthalene or pyrene, on anthracene chalcones (Zaini *et al.*, 2018 ▸), we have synthesized the title compound and report herein on its molecular and crystal structure, and optical properties.
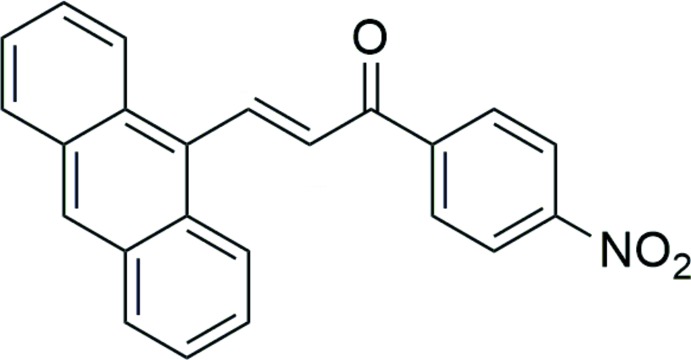


## Structural commentary   

**Figure 1:**
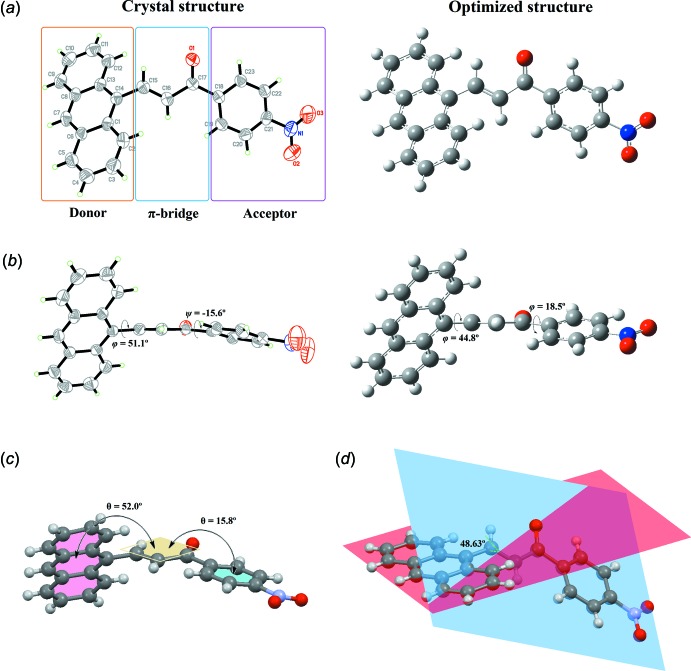
(*a*) The mol­ecular structure of the title compound based *D*–π–*A* system with displacement ellipsoids drawn at the 50% probability level and the optimized structure, (*b*) a representation of the twisted structures showing torsion angles, (*c*) and (*d*) the twisted structures showing dihedral angles.

The title chalcone compound consists of an anthracene ring system and a *para*-substituted nitro­benzene unit, representing a donor–π–acceptor (*D*–π–*A*) system (Fig. 1 ▸ *a*). The mol­ecular structure was optimized with the *Gaussian09W* software package (Frisch *et al.*, 2009 ▸) using the DFT method at the B3LYP/6-311G++(d,p) level of theory. All geometrical parameters calculated agree well with the experimental values. The compound adopts an *s-cis* conformation with respect to the C15=C16 [1.326 (5) Å; 1.347 (DFT) Å] and C17=O1 [1.232 (4) Å; 1.223 (DFT) Å] double bonds in the enone unit (C15=C16—C17=O1) and the structure is twisted around the C14—C15 bond with a C1—C14—C15—C16 torsion angle of 51.1 (6)° and slightly deviated around the C17—C18 bond with a C16—C17—C18—C19 torsion angle of −15.6 (5)°. The corresponding values by DFT are 44.8 and 18.5°, respectively (Fig. 1 ▸ *b*). These large twist angles are due to the bulkiness of the strong-electron-donor anthracene ring system (Zainuri *et al.*, 2018 ▸) and are also expected from the steric repulsion between the H atoms of the anthracene ring system and the ethyl­ene group. In addition, the enone unit [maximum deviation 0.020 (3) Å at C17] forms dihedral angles of 52.0 (2) and 15.8 (2)°, respectively, with the anthracene ring system [C1–C14, maximum deviation of 0.034 (4) Å at C5] and the nitro­benzene ring [C18–C23, maximum deviation 0.011 (4) Å at C20] (Fig. 1 ▸ *c*). Furthermore, a large dihedral angle of 48.63 (14)° is observed between the anthracene ring system and the nitro­benzene ring (Fig. 1 ▸ *d*); this could diminish the electronic effect between the two ring systems (Jung *et al.*, 2008 ▸).

## Supra­molecular features   

**Figure 2:**
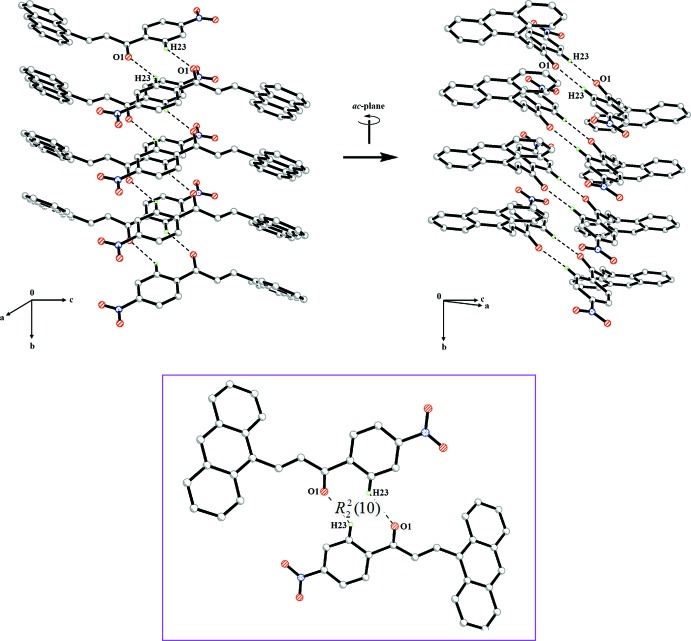
Packing diagrams of the title compound, showing C—H⋯O inter­actions (dashed lines).

In the crystal, the mol­ecules are linked *via* pairs of inter­molecular C—H⋯O inter­actions [C23—H23⋯O1^i^; symmetry code (i): −*x* + 1, −*y* + 2, −*z* + 1; Table 1 ▸), forming inversion dimers with an 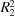
(10) graph-set motif. These dimers are stacked along the *b*-axis direction (Fig. 2 ▸).

**Figure 3:**
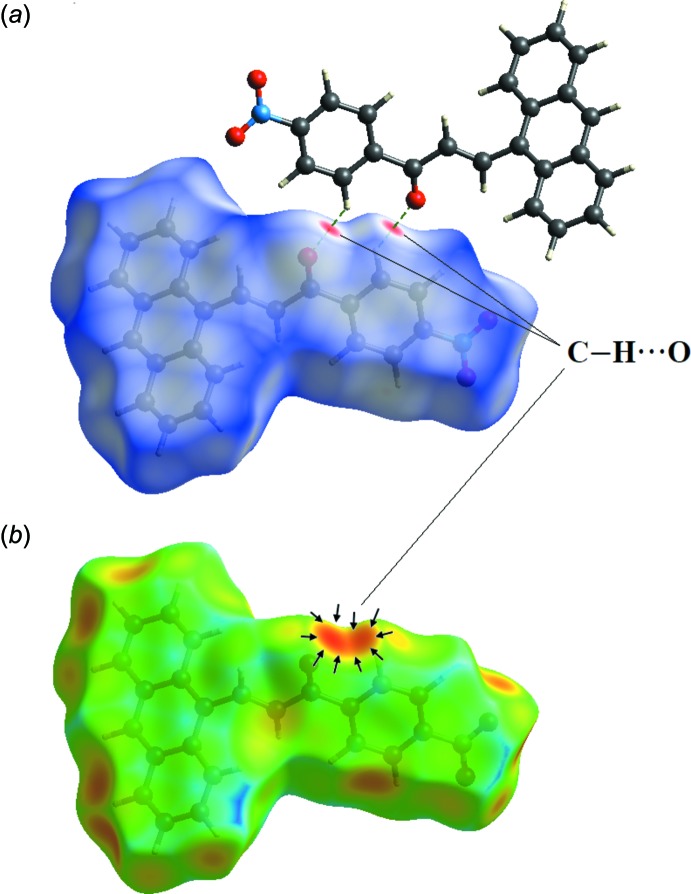
The Hirshfeld surfaces mapped over (*a*) *d*_norm_ and (*b*) *d*_e_, displaying the inter­molecular inter­actions.

**Figure 4:**
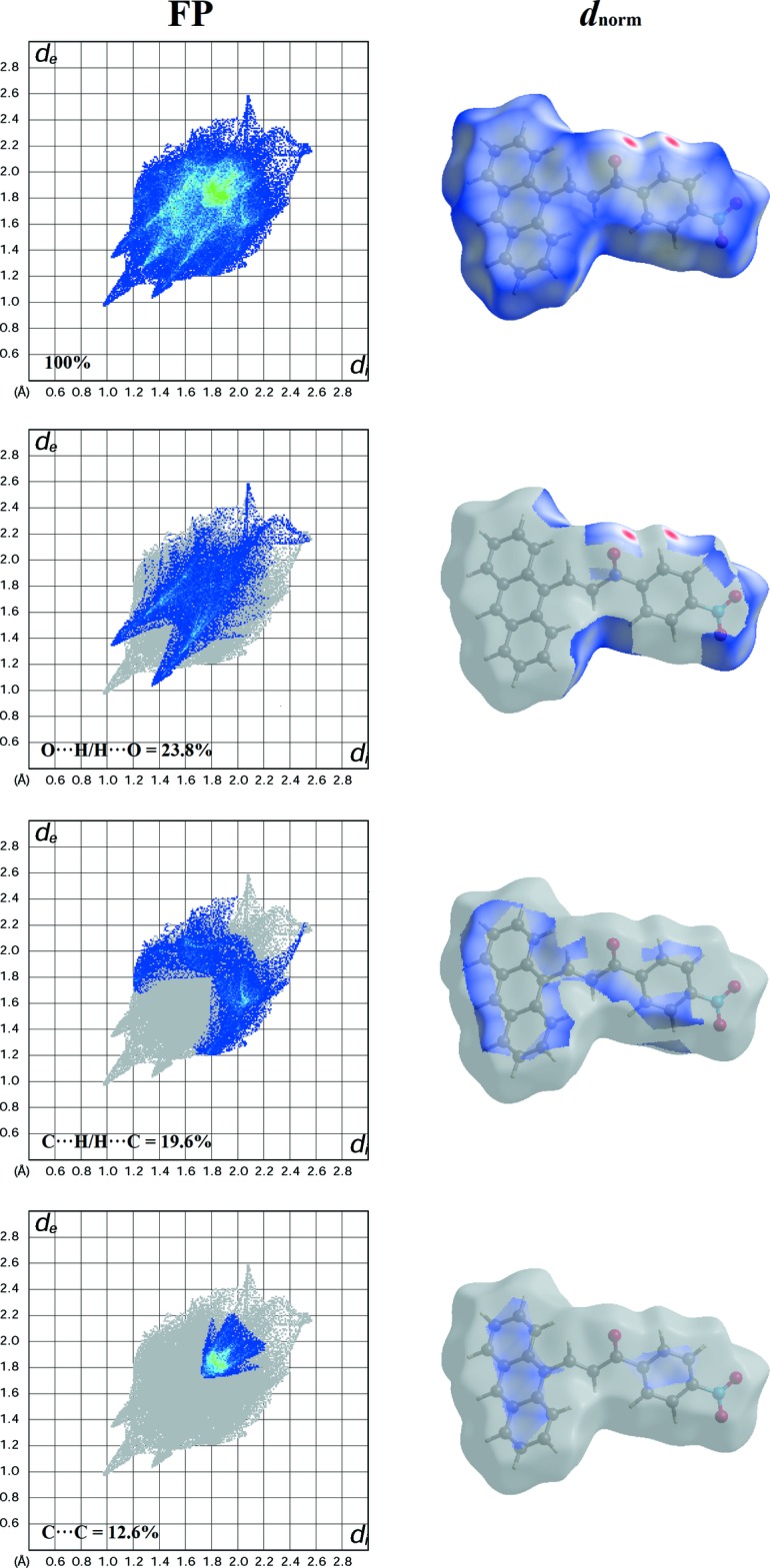
The fingerprint plots of the inter­molecular contacts with the corresponding *d*_norm_ surfaces, listing the percentage contributions to the total Hirshfeld surface.

The Hirshfeld surfaces and the related two dimensional fingerprint plots were generated using *Crystal Explorer*3.1 (Wolff *et al.*, 2012 ▸). The *d*_norm_ and *d*_e_ surfaces are presented in Fig. 3 ▸ *a* and Fig. 3 ▸ *b*, respectively. In the *d*_norm_ surface, the bright-red spots indicate the inter­molecular C—H⋯O inter­actions. These contacts are also confirmed by the pale-orange region marked with arrows in the *d*_e_ surface. The fingerprint plots (Ternavisk *et al.*, 2014 ▸) of the inter­molecular contacts with the corresponding *d*_norm_ surfaces (Fig. 4 ▸) show that the percentage contributions to the total Hirshfeld surface are 23.8, 19.6 and 12.6%, respectively, for the O⋯H/H⋯O, C⋯H/H⋯C and C⋯C contacts.

## UV–vis analysis and frontier mol­ecular orbitals   

**Figure 5:**
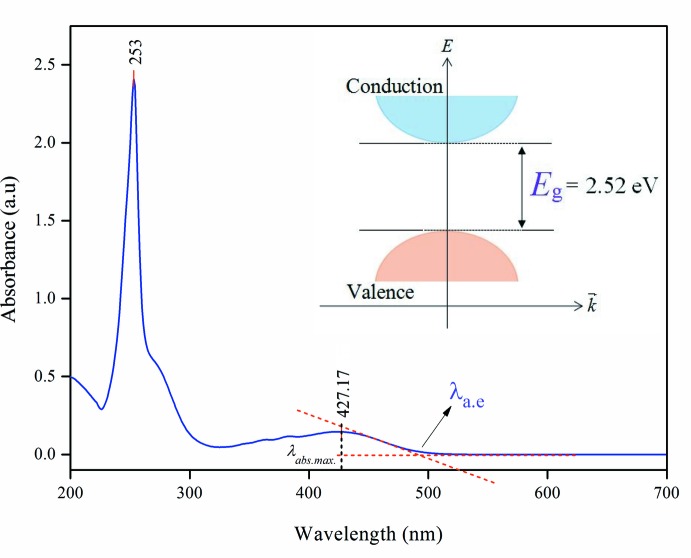
UV–vis spectrum of the title compound. Inset showed the experimental energy band gap obtained from absorption edge wavelength (λ_a.e_).

**Figure 6:**
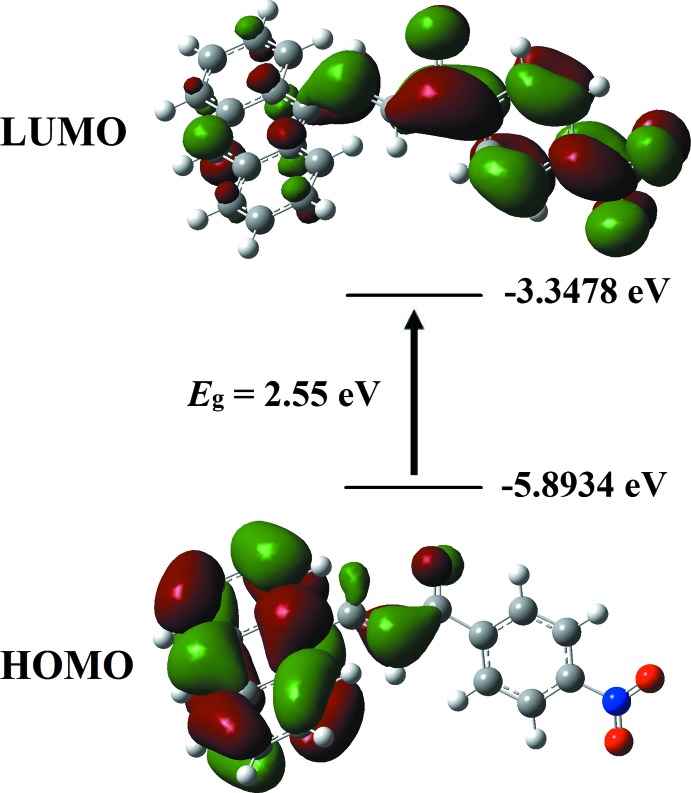
The spatial distributions of the HOMO and LUMO calculated for the title compound.

The measurement of the UV–vis absorption spectrum was carried out in an aceto­nitrile solution (10^−5^ *M*) with cut-off wavelength of 190 nm. Two major peaks at 253 and 427 nm were observed (Fig. 5 ▸). The strong band of 253 nm was assigned to the *n*–π^*^ transition. This sharp absorption peak arises due to the presence of carbonyl (C=O) and nitro substituent (NO_2_) functional groups (Zaini *et al.*, 2018 ▸). The energy band gap of 2.52 eV was evaluated from the UV–vis absorption edge (λ_a.e_) at 492.06 nm (Fig. 5 ▸). This small band-gap energy is suitable for optoelectronic applications as previously reported for the structure of chalcone (Prabhu *et al.*, 2016 ▸), and therefore exhibits a semiconducting nature (Rosencher & Vinter, 2002 ▸). The highest occupied mol­ecular orbital (HOMO) and the lowest unoccupied mol­ecular orbital (LUMO), known as frontier orbitals, obtained with the B3LYP/6-311G++(d,p) level calculation are illustrated in Fig. 6 ▸. The HOMO is mainly delocalized at the anthracene ring system. After excitation, the charge is localized at the enone and nitro­benzene moieties as depicted in the LUMO. The calculated HOMO–LUMO energy gap is 2.55 eV which is comparable with the UV–vis energy band gap obtained from the UV–vis absorption edge.

## Database survey   

A search of the Cambridge Structural Database (Version 5.40, last update February 2019; Groom *et al.*, 2016 ▸) revealed six closely related fused-ring chalcones, namely, *trans*-3-(9-anthr­yl)-1-(4-meth­oxy­phen­yl)prop-2-en-1-one (refcode EMULIT; Zhang *et al.*, 2016 ▸), 3-(anthracen-9-yl)-1-(4-chloro­phen­yl)prop-2-en-1-one (JAHPUG; Yu *et al.*, 2017 ▸), (*E*)-3-(anthracen-9-yl)-1-(4-bromo­phen­yl)prop-2-en-1-one (POP­BAY; Suwunwong *et al.*, 2009 ▸), (*Z*)-3-(anthracen-9-yl)-1-(2-eth­oxy­phen­yl)prop-2-en-1-one (KABHUS; Joothamongkhon *et al.*, 2010 ▸), (*E*)-3-(anthracen-9-yl)-1-(2-hy­droxy­phen­yl)prop-2-en-1-one (UNUDUD; Jasinski *et al.*, 2011 ▸; UNUDUD01; Chantrapromma *et al.*, 2011 ▸), (*E*)-3-(anthracen-9-yl)-1-(2-bromo­phen­yl)prop-2-en-1-one (WAFGOB; Fun *et al.*, 2010 ▸). Compounds EMULIT, JAHPUG and POPBAY are meth­oxy, chloro and bromo derivatives, respectively, substituted at the *para* position on the phenyl ring, while compounds KABHUS, UNUDUD (UNUDUD01) and WAFGOB are *ortho*-substituted eth­oxy, hy­droxy and bromo derivatives, respectively. Dihedral angles between the enone unit and the anthracene ring system and between the enone unit and the benzene ring are 81.6 (3) and 8.2 (4)°, respectively, for EMULIJ, 47.1 (3) and 22.9 (3)° for JAHPUG, 45.79 (10) and 20.88 (11)° for POPBAY, 82.49 (11) and 35.54 (13)° for KABHUS, 61.51 (9) and 14.56 (10)° [62.05 (9) and 11.04 (10)°] for UNUDUD, and 42.62 (16) and 63.00 (17)° for WAFGOB. The large dihedral angle of 82.49 (11)° between the enone unit and the anthracene ring system observed for KABHUS is due to the *Z* configuration of the mol­ecule. Inter­estingly, EMULIJ with an *E* configuration also shows a large dihedral angle of 81.6 (3)° between the enone unit and the anthracene ring system, whereas the dihedral angle between the enone unit and the benzene ring is extremely small [8.2 (4)°].

## Synthesis and crystallization   

A mixture of 4-nitro­aceto­phenone (0.5 mmol) and 9-anthracencarboxaldehyde (0.5 mmol) was dissolved in methanol (20 ml) and the solution stirred continuously. A catalytic amount of NaOH (5 ml, 20%) was added to the solution dropwise until a precipitate formed and the reaction was stirred continuously for about 5 h at room temperature. After stirring, the solution was poured into 60 ml of ice-cold distilled water. The resultant crude product was filtered and washed several times with with distilled water until the filtrate turned colourless. The dried precipitate was further recrystallized to obtain the corresponding chalcone. Red plate-shaped single crystals suitable for X-ray diffraction were obtained by slow evaporation of an acetone solution.

## Refinement   

Crystal data, data collection and structure refinement details are summarized in Table 2 ▸. The C-bound H atoms were placed in calculated positions (C—H = 0.93 Å) and were included in the refinement in the riding-model approximation, with *U*_iso_(H) = 1.2*U*_eq_(C). Four outliers (002), (420), (300) and (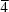
52) were omitted in the last cycle of refinement. The crystal used was a two-component merohedral twin (twin law 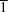 0 0 0 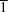 0 1 0 1). The refined ratio of the twin components was 0.1996 (16):0.8004 (16).

## Supplementary Material

Crystal structure: contains datablock(s) I, mo_MFZ5_w_0m. DOI: 10.1107/S2056989019005243/is5513sup1.cif

Structure factors: contains datablock(s) I. DOI: 10.1107/S2056989019005243/is5513Isup2.hkl

Comparison of bond lengths and angles between experimental and theoretical studies. DOI: 10.1107/S2056989019005243/is5513sup3.docx

Supporting information file. DOI: 10.1107/S2056989019005243/is5513Isup4.cml

CCDC reference: 1905274

Additional supporting information:  crystallographic information; 3D view; checkCIF report

## Figures and Tables

**Table 1:** Hydrogen-bond geometry (Å, °)

*D*—H⋯*A*	*D*—H	H⋯*A*	*D*⋯*A*	*D*—H⋯*A*
C23—H23*A*⋯O1^i^	0.93	2.49	3.240 (4)	138

Symmetry code: (i) 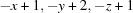.

**Table 2:** Experimental details

Crystal data	
Chemical formula	C_23_H_15_NO_3_
*M*_r_	353.36
Crystal system, space group	Monoclinic, *P*2_1_/*c*
Temperature (K)	296
*a*, *b*, *c* (Å)	10.8204 (10), 3.9364 (3), 40.420 (3)
β (°)	97.651 (3)
*V* (Å^3^)	1706.3 (2)
*Z*	4
Radiation type	Mo *K*α
μ (mm^−1^)	0.09
Crystal size (mm)	0.26 × 0.17 × 0.08

Data collection	
Diffractometer	Bruker APEXII CCD
Absorption correction	Multi-scan (*SADABS*; Bruker, 2009 ▸)
*T*_min_, *T*_max_	0.771, 0.970
No. of measured, independent and observed [*I* > 2σ(*I*)] reflections	45734, 3608, 2570
*R*_int_	0.113
(sin θ/λ)_max_ (Å^−1^)	0.617

Refinement	
*R*[*F* ^2^ > 2σ(*F* ^2^)], *wR*(*F* ^2^), *S*	0.074, 0.178, 1.09
No. of reflections	3608
No. of parameters	245
H-atom treatment	H-atom parameters constrained
Δρ_max_, Δρ_min_ (e Å^−3^)	0.20, −0.21

Computer programs: *APEX2* and *SAINT* (Bruker, 2009 ▸), *SHELXS97* and *SHELXTL* (Sheldrick, 2008 ▸) and *SHELXL2014* (Sheldrick, 2015 ▸).

## supplementary crystallographic information

### Crystal data

**Table:**

C_23_H_15_NO_3_	*F*(000) = 736
*M**_r_* = 353.36	*D*_x_ = 1.375 Mg m^−^^3^
Monoclinic, *P*2_1_/*c*	Mo *K*α radiation, λ = 0.71073 Å
*a* = 10.8204 (10) Å	Cell parameters from 9886 reflections
*b* = 3.9364 (3) Å	θ = 2.3–30.2°
*c* = 40.420 (3) Å	µ = 0.09 mm^−^^1^
β = 97.651 (3)°	*T* = 296 K
*V* = 1706.3 (2) Å^3^	Plate, red
*Z* = 4	0.26 × 0.17 × 0.08 mm

### Data collection

**Table:**

Bruker APEXII CCD diffractometer	2570 reflections with *I* > 2σ(*I*)
φ and ω scans	*R*_int_ = 0.113
Absorption correction: multi-scan (SADABS; Bruker, 2009)	θ_max_ = 26.0°, θ_min_ = 1.5°
*T*_min_ = 0.771, *T*_max_ = 0.970	*h* = −13→13
45734 measured reflections	*k* = −4→4
3608 independent reflections	*l* = −49→49

### Refinement

**Table:**

Refinement on *F*^2^	0 restraints
Least-squares matrix: full	Hydrogen site location: inferred from neighbouring sites
*R*[*F*^2^ > 2σ(*F*^2^)] = 0.074	H-atom parameters constrained
*wR*(*F*^2^) = 0.178	*w* = 1/[σ^2^(*F*_o_^2^) + (0.0651*P*)^2^ + 1.4385*P*], where *P* = (*F*_o_^2^ + 2*F*_c_^2^)/3
*S* = 1.09	(Δ/σ)_max_ < 0.001
3608 reflections	Δρ_max_ = 0.20 e Å^−^^3^
245 parameters	Δρ_min_ = −0.21 e Å^−^^3^

### Special details

**Table:**

Experimental. The following wavelength and cell were deduced by SADABS from the direction cosines etc. They are given here for emergency use only: CELL 0.71075 3.957 11.583 40.623 82.797 90.074 69.980
Geometry. All esds (except the esd in the dihedral angle between two l.s. planes) are estimated using the full covariance matrix. The cell esds are taken into account individually in the estimation of esds in distances, angles and torsion angles; correlations between esds in cell parameters are only used when they are defined by crystal symmetry. An approximate (isotropic) treatment of cell esds is used for estimating esds involving l.s. planes.
Refinement. Refined as a 2-component twin.

### Fractional atomic coordinates and isotropic or equivalent isotropic displacement parameters (Å^2^)

**Table:**

	*x*	*y*	*z*	*U*_iso_*/*U*_eq_	
O1	0.4171 (2)	0.9048 (7)	0.44992 (6)	0.0512 (7)	
O2	0.0772 (4)	0.1119 (11)	0.57047 (8)	0.0950 (12)	
O3	0.2506 (4)	0.2855 (14)	0.59652 (8)	0.1070 (16)	
N1	0.1793 (4)	0.2508 (11)	0.57135 (9)	0.0638 (10)	
C1	0.1551 (3)	0.5116 (9)	0.33952 (8)	0.0394 (9)	
C2	0.0565 (4)	0.6557 (11)	0.35514 (8)	0.0487 (10)	
H2A	0.0749	0.7540	0.3761	0.058*	
C3	−0.0635 (4)	0.6530 (12)	0.34009 (9)	0.0572 (11)	
H3A	−0.1260	0.7449	0.3511	0.069*	
C4	−0.0939 (4)	0.5133 (13)	0.30826 (10)	0.0601 (12)	
H4A	−0.1765	0.5128	0.2983	0.072*	
C5	−0.0048 (4)	0.3793 (11)	0.29185 (9)	0.0527 (10)	
H5A	−0.0269	0.2860	0.2708	0.063*	
C6	0.1232 (3)	0.3783 (10)	0.30643 (8)	0.0420 (9)	
C7	0.2156 (4)	0.2487 (10)	0.28935 (8)	0.0469 (9)	
H7A	0.1936	0.1626	0.2679	0.056*	
C8	0.3404 (4)	0.2438 (10)	0.30333 (8)	0.0433 (9)	
C9	0.4357 (4)	0.1017 (11)	0.28588 (9)	0.0537 (10)	
H9A	0.4140	0.0136	0.2645	0.064*	
C10	0.5562 (4)	0.0937 (12)	0.29994 (10)	0.0598 (11)	
H10A	0.6172	0.0086	0.2880	0.072*	
C11	0.5891 (4)	0.2144 (12)	0.33262 (10)	0.0604 (11)	
H11A	0.6717	0.2002	0.3424	0.072*	
C12	0.5027 (3)	0.3513 (11)	0.35013 (9)	0.0513 (10)	
H12A	0.5275	0.4338	0.3715	0.062*	
C13	0.3747 (3)	0.3710 (9)	0.33633 (8)	0.0399 (8)	
C14	0.2818 (3)	0.5076 (9)	0.35409 (8)	0.0377 (8)	
C15	0.3204 (3)	0.6376 (9)	0.38796 (8)	0.0423 (9)	
H15A	0.3855	0.7937	0.3901	0.051*	
C16	0.2745 (4)	0.5611 (9)	0.41586 (8)	0.0424 (9)	
H16A	0.2055	0.4190	0.4149	0.051*	
C17	0.3311 (3)	0.6979 (9)	0.44821 (8)	0.0367 (8)	
C18	0.2866 (3)	0.5810 (8)	0.47993 (7)	0.0345 (8)	
C19	0.1747 (3)	0.4084 (10)	0.48049 (8)	0.0447 (9)	
H19A	0.1239	0.3599	0.4606	0.054*	
C20	0.1392 (4)	0.3096 (10)	0.51059 (9)	0.0481 (10)	
H20A	0.0636	0.1987	0.5111	0.058*	
C21	0.2152 (3)	0.3743 (10)	0.53969 (8)	0.0433 (9)	
C22	0.3264 (3)	0.5453 (10)	0.53986 (8)	0.0448 (9)	
H22A	0.3775	0.5890	0.5598	0.054*	
C23	0.3599 (3)	0.6495 (10)	0.50987 (8)	0.0412 (9)	
H23A	0.4338	0.7694	0.5097	0.049*	

### Atomic displacement parameters (Å^2^)

**Table:**

	*U*^11^	*U*^22^	*U*^33^	*U*^12^	*U*^13^	*U*^23^
O1	0.0545 (16)	0.0532 (16)	0.0449 (13)	−0.0130 (15)	0.0028 (12)	−0.0031 (13)
O2	0.087 (2)	0.121 (3)	0.083 (2)	−0.033 (3)	0.0344 (19)	0.006 (2)
O3	0.095 (3)	0.178 (5)	0.0471 (17)	−0.025 (3)	0.0076 (18)	0.017 (2)
N1	0.069 (2)	0.073 (3)	0.053 (2)	−0.001 (2)	0.0200 (19)	0.0001 (19)
C1	0.051 (2)	0.0347 (19)	0.0338 (17)	0.0008 (17)	0.0093 (16)	0.0037 (15)
C2	0.058 (3)	0.051 (2)	0.0376 (18)	0.007 (2)	0.0070 (18)	0.0028 (18)
C3	0.052 (2)	0.067 (3)	0.053 (2)	0.013 (2)	0.0126 (19)	0.012 (2)
C4	0.050 (2)	0.073 (3)	0.055 (2)	0.002 (2)	−0.003 (2)	0.013 (2)
C5	0.062 (3)	0.053 (2)	0.041 (2)	−0.007 (2)	−0.0009 (19)	0.0046 (19)
C6	0.051 (2)	0.041 (2)	0.0337 (17)	−0.0066 (19)	0.0058 (16)	0.0053 (16)
C7	0.069 (3)	0.039 (2)	0.0327 (17)	−0.003 (2)	0.0071 (18)	−0.0038 (16)
C8	0.058 (2)	0.0345 (19)	0.0385 (18)	0.0002 (19)	0.0109 (18)	0.0030 (16)
C9	0.067 (3)	0.049 (2)	0.047 (2)	0.002 (2)	0.019 (2)	−0.0030 (19)
C10	0.062 (3)	0.055 (3)	0.068 (3)	0.009 (2)	0.028 (2)	0.004 (2)
C11	0.050 (2)	0.062 (3)	0.070 (3)	0.002 (2)	0.012 (2)	0.007 (2)
C12	0.051 (2)	0.054 (3)	0.049 (2)	0.001 (2)	0.0065 (19)	0.0017 (19)
C13	0.050 (2)	0.0312 (18)	0.0397 (18)	0.0006 (18)	0.0097 (16)	0.0052 (16)
C14	0.047 (2)	0.0307 (19)	0.0352 (17)	−0.0004 (17)	0.0048 (15)	0.0031 (15)
C15	0.048 (2)	0.0350 (19)	0.0425 (18)	−0.0003 (18)	0.0015 (16)	−0.0009 (16)
C16	0.052 (2)	0.0357 (19)	0.0382 (18)	0.0012 (19)	0.0019 (16)	0.0003 (16)
C17	0.035 (2)	0.0345 (19)	0.0395 (18)	0.0067 (18)	0.0008 (15)	−0.0026 (15)
C18	0.0365 (19)	0.0290 (17)	0.0370 (17)	0.0099 (16)	0.0013 (14)	−0.0049 (15)
C19	0.040 (2)	0.050 (2)	0.0421 (19)	0.0021 (19)	−0.0001 (16)	−0.0099 (18)
C20	0.044 (2)	0.047 (2)	0.055 (2)	−0.0051 (19)	0.0129 (18)	−0.0060 (19)
C21	0.047 (2)	0.045 (2)	0.0392 (18)	0.011 (2)	0.0118 (16)	−0.0021 (17)
C22	0.042 (2)	0.054 (2)	0.0371 (18)	0.007 (2)	0.0015 (15)	−0.0059 (17)
C23	0.039 (2)	0.044 (2)	0.0396 (18)	0.0033 (18)	0.0036 (15)	−0.0052 (17)

### Geometric parameters (Å, º)

**Table:**

O1—C17	1.232 (4)	C10—H10A	0.9300
O2—N1	1.229 (5)	C11—C12	1.358 (5)
O3—N1	1.200 (4)	C11—H11A	0.9300
N1—C21	1.468 (5)	C12—C13	1.424 (5)
C1—C14	1.418 (5)	C12—H12A	0.9300
C1—C2	1.427 (5)	C13—C14	1.417 (5)
C1—C6	1.435 (5)	C14—C15	1.469 (5)
C2—C3	1.359 (5)	C15—C16	1.326 (5)
C2—H2A	0.9300	C15—H15A	0.9300
C3—C4	1.398 (6)	C16—C17	1.470 (5)
C3—H3A	0.9300	C16—H16A	0.9300
C4—C5	1.348 (6)	C17—C18	1.500 (5)
C4—H4A	0.9300	C18—C23	1.382 (4)
C5—C6	1.431 (5)	C18—C19	1.392 (5)
C5—H5A	0.9300	C19—C20	1.379 (5)
C6—C7	1.386 (5)	C19—H19A	0.9300
C7—C8	1.393 (5)	C20—C21	1.366 (5)
C7—H7A	0.9300	C20—H20A	0.9300
C8—C13	1.427 (5)	C21—C22	1.378 (5)
C8—C9	1.438 (5)	C22—C23	1.373 (5)
C9—C10	1.352 (6)	C22—H22A	0.9300
C9—H9A	0.9300	C23—H23A	0.9300
C10—C11	1.404 (6)		
			
O3—N1—O2	123.3 (4)	C11—C12—C13	121.2 (4)
O3—N1—C21	119.1 (4)	C11—C12—H12A	119.4
O2—N1—C21	117.6 (4)	C13—C12—H12A	119.4
C14—C1—C2	123.9 (3)	C14—C13—C12	122.7 (3)
C14—C1—C6	118.8 (3)	C14—C13—C8	119.5 (3)
C2—C1—C6	117.2 (3)	C12—C13—C8	117.8 (3)
C3—C2—C1	121.6 (3)	C13—C14—C1	120.4 (3)
C3—C2—H2A	119.2	C13—C14—C15	118.1 (3)
C1—C2—H2A	119.2	C1—C14—C15	121.5 (3)
C2—C3—C4	120.6 (4)	C16—C15—C14	128.4 (4)
C2—C3—H3A	119.7	C16—C15—H15A	115.8
C4—C3—H3A	119.7	C14—C15—H15A	115.8
C5—C4—C3	120.7 (4)	C15—C16—C17	121.0 (4)
C5—C4—H4A	119.6	C15—C16—H16A	119.5
C3—C4—H4A	119.6	C17—C16—H16A	119.5
C4—C5—C6	121.0 (4)	O1—C17—C16	120.9 (3)
C4—C5—H5A	119.5	O1—C17—C18	118.7 (3)
C6—C5—H5A	119.5	C16—C17—C18	120.3 (3)
C7—C6—C5	121.2 (3)	C23—C18—C19	118.7 (3)
C7—C6—C1	119.9 (3)	C23—C18—C17	118.5 (3)
C5—C6—C1	118.8 (3)	C19—C18—C17	122.8 (3)
C6—C7—C8	121.8 (3)	C20—C19—C18	119.8 (3)
C6—C7—H7A	119.1	C20—C19—H19A	120.1
C8—C7—H7A	119.1	C18—C19—H19A	120.1
C7—C8—C13	119.5 (3)	C21—C20—C19	120.1 (4)
C7—C8—C9	121.8 (3)	C21—C20—H20A	120.0
C13—C8—C9	118.7 (3)	C19—C20—H20A	120.0
C10—C9—C8	121.2 (4)	C20—C21—C22	121.3 (3)
C10—C9—H9A	119.4	C20—C21—N1	119.3 (4)
C8—C9—H9A	119.4	C22—C21—N1	119.3 (3)
C9—C10—C11	119.8 (4)	C23—C22—C21	118.3 (3)
C9—C10—H10A	120.1	C23—C22—H22A	120.9
C11—C10—H10A	120.1	C21—C22—H22A	120.9
C12—C11—C10	121.2 (4)	C22—C23—C18	121.8 (4)
C12—C11—H11A	119.4	C22—C23—H23A	119.1
C10—C11—H11A	119.4	C18—C23—H23A	119.1
			
C14—C1—C2—C3	180.0 (4)	C8—C13—C14—C15	−179.7 (3)
C6—C1—C2—C3	3.1 (6)	C2—C1—C14—C13	−177.5 (3)
C1—C2—C3—C4	−1.3 (7)	C6—C1—C14—C13	−0.6 (5)
C2—C3—C4—C5	−0.1 (7)	C2—C1—C14—C15	3.6 (6)
C3—C4—C5—C6	−0.4 (7)	C6—C1—C14—C15	−179.5 (3)
C4—C5—C6—C7	−178.4 (4)	C13—C14—C15—C16	−127.8 (4)
C4—C5—C6—C1	2.3 (6)	C1—C14—C15—C16	51.1 (6)
C14—C1—C6—C7	0.1 (5)	C14—C15—C16—C17	175.5 (3)
C2—C1—C6—C7	177.1 (3)	C15—C16—C17—O1	5.0 (5)
C14—C1—C6—C5	179.4 (3)	C15—C16—C17—C18	−173.6 (3)
C2—C1—C6—C5	−3.5 (5)	O1—C17—C18—C23	−13.9 (5)
C5—C6—C7—C8	−179.5 (4)	C16—C17—C18—C23	164.7 (3)
C1—C6—C7—C8	−0.2 (6)	O1—C17—C18—C19	165.7 (3)
C6—C7—C8—C13	0.9 (6)	C16—C17—C18—C19	−15.6 (5)
C6—C7—C8—C9	178.5 (4)	C23—C18—C19—C20	−0.1 (5)
C7—C8—C9—C10	−179.0 (4)	C17—C18—C19—C20	−179.7 (3)
C13—C8—C9—C10	−1.4 (6)	C18—C19—C20—C21	−1.4 (6)
C8—C9—C10—C11	2.4 (7)	C19—C20—C21—C22	1.6 (6)
C9—C10—C11—C12	−2.4 (7)	C19—C20—C21—N1	−176.7 (4)
C10—C11—C12—C13	1.4 (7)	O3—N1—C21—C20	174.4 (4)
C11—C12—C13—C14	179.0 (4)	O2—N1—C21—C20	−4.7 (6)
C11—C12—C13—C8	−0.4 (6)	O3—N1—C21—C22	−3.9 (6)
C7—C8—C13—C14	−1.5 (5)	O2—N1—C21—C22	177.1 (4)
C9—C8—C13—C14	−179.1 (3)	C20—C21—C22—C23	−0.2 (6)
C7—C8—C13—C12	178.0 (4)	N1—C21—C22—C23	178.1 (3)
C9—C8—C13—C12	0.3 (5)	C21—C22—C23—C18	−1.4 (5)
C12—C13—C14—C1	−178.1 (4)	C19—C18—C23—C22	1.5 (5)
C8—C13—C14—C1	1.3 (5)	C17—C18—C23—C22	−178.8 (3)
C12—C13—C14—C15	0.8 (5)		

### Hydrogen-bond geometry (Å, º)

**Table:**

*D*—H···*A*	*D*—H	H···*A*	*D*···*A*	*D*—H···*A*
C23—H23*A*···O1^i^	0.93	2.49	3.240 (4)	138

Symmetry code: (i) −*x*+1, −*y*+2, −*z*+1.
