# Supplementary material for: Crystal structure and optical properties of fused-ring chalcone (E)-3-(anthracen-9-yl)-1-(4-nitro­phen­yl)prop-2-en-1-one
Source: Acta Crystallogr E Crystallogr Commun. 2019 Apr 25;75(Pt 5):685–9. doi: 10.1107/S2056989019005243 (PMC6505597; doi:10.1107/S2056989019005243)
Supplement: Supplementary file 3 [file e-75-00685-sup3.docx]

**Supplementary Information**

Table S1: Comparison of bond lengths and angles between experimental and theoretical studies

| **Bond Distance (Å)** | | |
| --- | --- | --- |
| Atoms | Experimental | DFT B3LYP/6-311 G++(d,p) |
| O2 - N1 | 1.229 (5) | 1.224 |
| O3 - N1 | 1.200 (4) | 1.224 |
| O1 - C17 | 1.232 (4) | 1.223 |
| C14 - C15 | 1.469 (5) | 1.465 |
| C15 - C16 | 1.326 (5) | 1.347 |
| C16 - C17 | 1.470 (5) | 1.480 |
| C17 - C18 | 1.500 (5) | 1.509 |

| Bond Angle (°) | | |
| --- | --- | --- |
| Atoms | Experimental | DFT B3LYP/6-311 G++(d,p) |
| O1 - C17 - C18 | 118.7 (3) | 119.3 |
| O1 - C17 - C16 | 120.9 (3) | 122.1 |
| C1 - C14 - C15 | 121.5 (3) | 122.3 |
| C13 - C14 - C15 | 118.1 (3) | 117.8 |
| C14 - C15 - C16 | 128.4 (4) | 127.6 |
| C15 - C16 - C17 | 121.0 (4) | 120.3 |
| C16 - C17 - C18 | 120.3 (3) | 118.6 |
| C17 - C18 - C23 | 118.5 (3) | 117.6 |
| C17 - C18 - C19 | 122.8 (3) | 123.2 |
| O2 - N1 - O3 | 123.3 (4) | 124.8 |
| C21 - N1 - O3 | 119.1 (4) | 117.6 |
| C21 - N1 - O2 | 117.6 (4) | 117.6 |

| Torsion Angle (°) | | |
| --- | --- | --- |
| Atoms | Experimental | DFT B3LYP/6-311 G++(d,p) |
| C15 - C16 - C17 - O1 | 5.0 (5) | 8.2 |
| O1 - C17 - C18 - C23 | −13.9 (5) | 16.5 |
| O1 - C17 - C18 - C19 | 165.7 (3) | -162.2 |
| C1 - C14 - C15 - C16 | 51.1 (6) | 44.8 |
| C13 - C14 - C15 - C16 | −127.8 (4) | -136.0 |
| C14 - C15 - C16 - C17 | 175.5 (3) | 179.4 |
| C15 - C16 - C17 - C18 | −173.6 (3) | -172.5 |
| C16 - C17 - C18 - C19 | −15.6 (5) | 18.5 |
| C16 - C17 - C18 - C23 | 164.7 (3) | -162.9 |
